# BCG induced lower urinary tract symptoms during treatment for NMIBC—Mechanisms and management strategies

**DOI:** 10.3389/fnins.2023.1327053

**Published:** 2024-01-08

**Authors:** Georgia Bourlotos, William Baigent, Matthew Hong, Sophie Plagakis, Luke Grundy

**Affiliations:** ^1^College of Medicine and Public Health, Flinders Health and Medical Research Institute, Flinders University, Bedford Park, SA, Australia; ^2^Urology Unit, Flinders Medical Centre, Bedford Park, SA, Australia

**Keywords:** Bacillus Calmette-Guérin (BCG), cystitis, NMIBC, LUTS, afferent sensitization, bladder cancer (BCa), pelvic pain

## Abstract

Non-muscle invasive bladder cancer (NMIBC) accounts for ~70–75% of total bladder cancer tumors and requires effective early intervention to avert progression. The cornerstone of high-risk NMIBC treatment involves trans-urethral resection of the tumor followed by intravesical Bacillus Calmette-Guerin (BCG) immunotherapy. However, BCG therapy is commonly accompanied by significant lower urinary tract symptoms (LUTS) including urinary urgency, urinary frequency, dysuria, and pelvic pain which can undermine treatment adherence and clinical outcomes. Despite this burden, the mechanisms underlying the development of BCG-induced LUTS have yet to be characterized. This review provides a unique perspective on the mechanisms thought to be responsible for the development of BCG-induced LUTS by focussing on the sensory nerves responsible for bladder sensory transduction. This review focuses on how the physiological response to BCG, including inflammation, urothelial permeability, and direct interactions between BCG and sensory nerves could drive bladder afferent sensitization leading to the development of LUTS. Additionally, this review provides an up-to-date summary of the latest clinical data exploring interventions to relieve BCG-induced LUTS, including therapeutic targeting of bladder contractions, inflammation, increased bladder permeability, and direct inhibition of bladder sensory signaling. Addressing the clinical burden of BCG-induced LUTS holds significant potential to enhance patient quality of life, treatment compliance, and overall outcomes in NMIBC management. However, the lack of knowledge on the pathophysiological mechanisms that drive BCG-induced LUTS has limited the development of novel and efficacious therapeutic options. Further research is urgently required to unravel the mechanisms that drive BCG-induced LUTS.

## Introduction

The majority (~70–75%) of bladder cancer tumors are found within the urothelium and lamina propria and are classified as non-muscle invasive bladder cancers (NMIBC) (van den Bosch and Alfred Witjes, 2011). The gold standard treatment for patients with intermediate- or high-risk NMIBC is trans-urethral resection of the bladder tumor (TURBT) followed by intravesical instillations of Bacillus Calmette-Guerin (BCG) (Lobo et al., 2021; Thyavihally et al., 2022).

BCG immunotherapy is the most successful adjuvant therapy for reducing the rate of NMIBC recurrence and progression (Kamat et al., 2015; Jiang and Redelman-Sidi, 2022; Unsworth-White et al., 2022). However, approximately 70% of patients undergoing BCG immunotherapy will develop lower urinary tract side effects characterized by sensory dysfunction including urinary urgency, urinary frequency, dysuria, and bladder pain that significantly impact wellbeing during treatment (Alexandroff et al., 1999; Brausi et al., 2014; Nouhaud et al., 2017; Danielsson et al., 2018; Unsworth-White et al., 2022; Yuen et al., 2022). Moreover, ~7–20% of patients are forced to discontinue BCG therapy due to the severity of their lower urinary tract symptoms (LUTS), significantly increasing the risk of cancer progression and reducing survival (van der Meijden et al., 2003; Brausi et al., 2014; Krajewski et al., 2018; Lebacle et al., 2021). Despite this clinical significance, the mechanisms underlying BCG-induced LUTS have yet to be determined, and there are no clear guidelines for their prevention or treatment.

**Figure 1:**
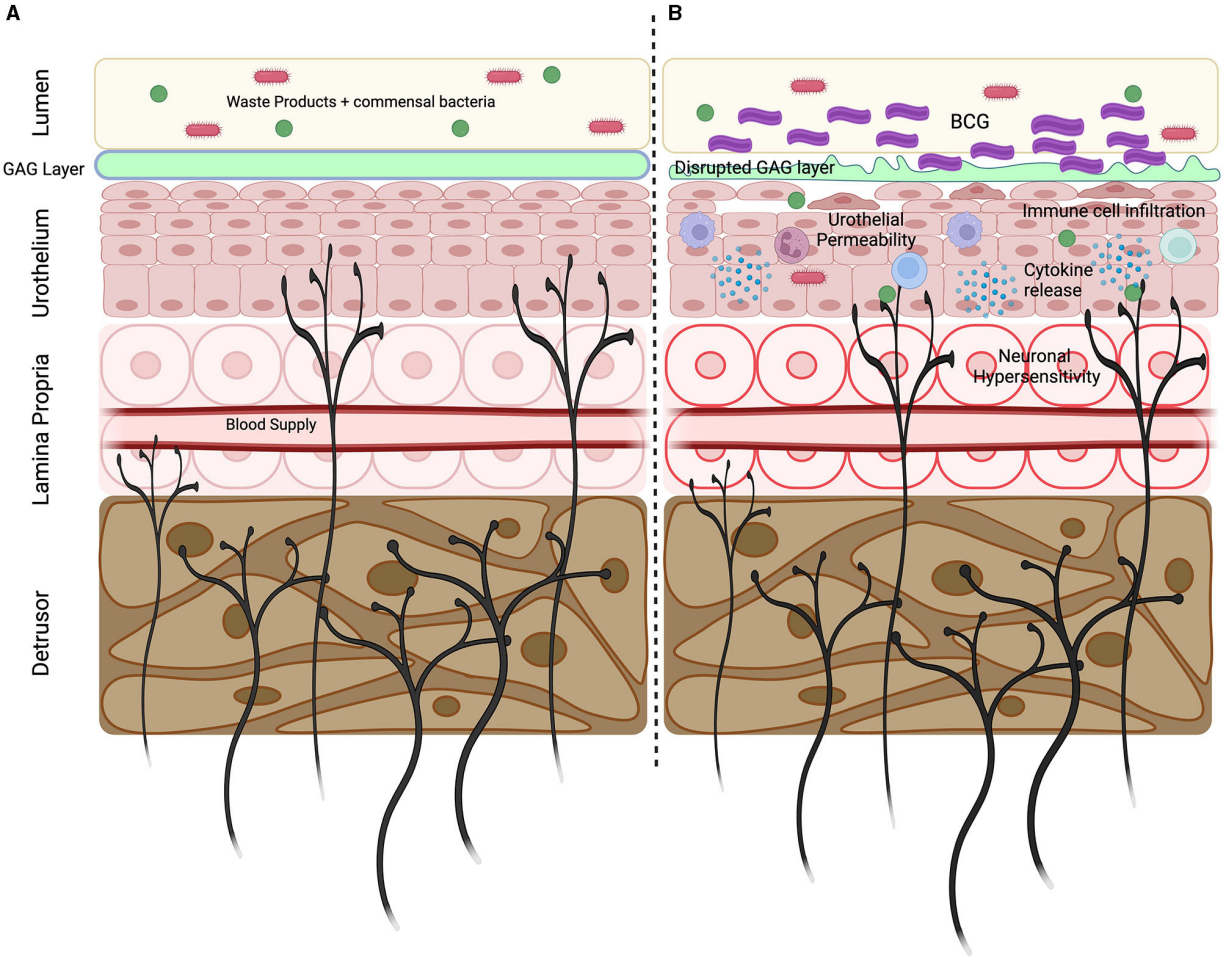
Key mechanisms underlying BCG induced LUTS. **(A)** Sensory nerves innervate throughout the bladder wall with peripheral endings located within the detrusor, lamina propria and urothelium. Sensory nerves express unique repertoires of ion channels and receptors allowing them to detect bladder stretch and respond to pathogens, inflammatory mediators, and the products of cellular destruction. Bladder sensory nerves are vital in detecting bladder damage and relaying information regarding bladder state. **(B)** Intravesical BCG infusions initiates an inflammatory response characterized by the immune cell infiltration and the release of cytokines. Inflammation promotes GAG later disruption and increased urothelial permeability, allowing urine and commensal bacteria to enter the bladder wall. Urinary solutes and inflammatory mediators can sensitize the peripheral endings of sensory nerves to induce neuronal hypersensitivity that precipitates the development of LUTS. Created using Biorender.com.

Normal bladder sensation is determined by the intensity of the afferent signal transduced from the bladder wall into the central nervous system (Fowler et al., 2008; Spencer et al., 2018) (Figure 1). Bladder afferents express mechanosensitive ion channels that respond to subtle changes in bladder stretch during normal bladder filling, relaying information regarding the fill state of the bladder to the spinal cord (Groat and Yoshimura, 2009; Grundy et al., 2019). Within the spinal cord, the terminal ends of bladder afferents trigger subconscious efferent reflexes that regulate detrusor and urethral function to maintain urine storage and continence during the filling phase of the micturition cycle (Fowler et al., 2008; Groat and Yoshimura, 2009). As the bladder continues to fill, the intensity of the sensory signal from the bladder increases, and this translates into a progressive increase in bladder sensations from fullness toward discomfort and pain (Groat and Yoshimura, 2009; Grundy et al., 2018a, 2019). Healthy bladder function relies on the integration of afferent signals with excitatory and inhibitory inputs from the anterior cingulate cortex, insula, hypothalamus, and prefrontal cortex to provide an overall assessment of the appropriateness to urinate (Fowler et al., 2008; Groat and Yoshimura, 2009; Grundy et al., 2018a). Consequentially, if bladder afferents become sensitized, such that exaggerated afferent signaling occurs during normal bladder filling, this can have profound impacts on bladder sensation and function, including the emergence of lower urinary tract symptoms (LUTS). Bladder sensory nerves can be sensitized by a range of factors, including infection, inflammation, and bladder damage, representing a common mechanism underlying the occurrence of LUTS in various bladder-related conditions, including urinary tract infections, overactive bladder syndrome, interstitial cystitis/bladder pain syndrome (IC/BPS), and cystitis induced by chemotherapy (Groat and Yoshimura, 2009; Grundy et al., 2018a; Mills et al., 2020). As patients with BCG-induced LUTS experience similar undesirable bladder sensations, it is highly likely that exaggerated sensory signaling from the bladder is a major contributing factor.

This review summarizes the latest preclinical and clinical research to highlight the pathophysiological mechanisms that are thought to underlie afferent sensitization during BCG immunotherapy for NMIBC. Additionally, we discuss the clinical interventions available that target these mechanisms and have shown promise in improving patient quality of life and immunotherapy persistence.

## Non-muscle invasive bladder cancer (NMIBC)

Bladder cancer is a lethal and diverse disease of the bladder, ranging from non-invasive tumors to advanced-stage malignancies (Saginala et al., 2020; Halaseh et al., 2022). Bladder cancer is currently the tenth most diagnosed cancer in the world (Deng-Xiong et al., 2022; Halaseh et al., 2022), predominantly impacts individuals over the age of 55 (Saginala et al., 2020; Halaseh et al., 2022), and results in approximately 200,000 deaths each year (Pettenati and Ingersoll, 2018; Saginala et al., 2020).

NMIBC tumors are localized within the superficial layers of the bladder wall, including the lamina propria and the urothelium, and account for ~70–75% of all bladder cancers (Cassell et al., 2019). In the absence of adequate therapeutic options, approximately 20% of patients with NMIBC tumors develop muscle invasive bladder cancer (MIBC) (van den Bosch and Alfred Witjes, 2011). MIBC has a significantly higher mortality rate than NMIBC (Patel et al., 2020), highlighting the importance of early detection and effective treatment of NMIBC.

## BCG treatment for NMIBC

Most patients presenting with NMIBC will undergo trans-urethral resection of bladder tumor (TURBT). For patients with intermediate- or high-risk NMIBC, a 6 week course of intravesical Bacillus Calmette-Guerin (BCG)-based immunotherapy is the gold standard adjuvant therapy (Nargund et al., 2012). Weekly BCG via intravesical instillation starting two weeks post TURBT demonstrates a 55–75% success rate in preventing tumor recurrence depending on cancer stage (Nargund et al., 2012; Tang and Chang, 2015; Pettenati and Ingersoll, 2018; Thyavihally et al., 2022). For patients that initially respond to BCG, maintenance therapy is recommended to ensure tumor suppression (Lamm et al., 2000). Specific maintenance schedules vary across countries and health care settings, but individuals will commonly follow the SWOG protocol, which requires 3 weekly installations of BCG at 3 and 6 months post induction and then every 6 months up to 3 years (Kamat et al., 2015).

## Lower urinary tract side effects of BCG therapy

Whilst the efficacy of BCG in the treatment of NMIBC is well-established, BCG instillations commonly cause local and systemic side effects that present complications in patient treatment. BCG induced lower urinary tract symptoms (LUTS), including dysuria (painful urination), severe urinary urgency and frequency, and pelvic pain are reported in ~70–80% of patients in the initial 48 h following each infusion and can be debilitating (Alexandroff et al., 1999; Green et al., 2018; Guallar-Garrido and Julián, 2020; Unsworth-White et al., 2022). As many as 20% of patients must discontinue BCG treatment due to the severity of their LUTS, with no reported sex-based differences in BCG tolerability (Liu et al., 2019; Sharma et al., 2020; Fadel et al., 2022). Sadly, however, there is a significant association between early cessation of BCG treatment and cancer progression and reduced survival (van der Meijden et al., 2003; Lerner et al., 2009; Zlotta et al., 2009; Brausi et al., 2014; Kamat et al., 2016; Krajewski et al., 2018; Lebacle et al., 2021). Consequently, patients are more likely to undergo radical cystectomy (bladder removal) and endure life-long changes in quality of life (Aldousari and Kassouf, 2010; Waked et al., 2020). An additional often overlooked cohort of BCG treated patients will also develop chronic LUTS, encompassing increased urinary urgency, urinary frequency, cystitis, pelvic pain, and dysuria that persists well beyond the initial completion or cessation of BCG treatment (Nargund et al., 2012; Imperatore et al., 2018; Liu et al., 2019; Sharma et al., 2020).

## Mechanisms underlying BCG induced LUTS

The mechanisms underlying BCG-induced LUTS have yet to be determined, and multiple elements likely compound. However, the sensory nature of the symptoms, and the similarities to LUTS that are present in patients with interstitial cystitis/bladder pain syndrome and acute urinary tract infection implicate exaggerated sensory signaling from the bladder as a major contributing factor.

### Sensitisation of bladder afferents

Exaggerated sensory signaling occurs following peripheral afferent sensitization. Afferent sensitization commonly arises during injury or illness, when sensory afferents are exposed to molecular factors that increase their excitability including bacteria, inflammatory mediators, and the products of cellular destruction (Chu et al., 2020). Afferent sensitization plays a fundamental role in the physiological healing response, delivering essential sensory feedback to modify behavior and support tissue repair (Baral et al., 2019). Bladder afferents can be sensitized by exposure to a variety of inflammatory mediators including histamine, cytokines, prostaglandins, nerve growth factor and serotonin (Groat and Yoshimura, 2009; Schnegelsberg et al., 2010; Grundy et al., 2018a, 2020a; Konthapakdee et al., 2019; Brierley et al., 2020). Furthermore, many inflammatory mediators also recruit “silent afferents” to become mechanically sensitive, further increasing peripheral drive from the bladder to the central nervous system during normal bladder function (Groat and Yoshimura, 2009; Brierley et al., 2020; Grundy et al., 2020a). Recent studies have shown that bladder afferents can also be sensitized directly by bacteria, their toxins and metabolites, and the toxic contents of urine following urothelial barrier breakdown (Groat and Yoshimura, 2009; Grundy et al., 2018a, 2020b; Brierley et al., 2020; Montalbetti et al., 2022). Consequently, during pathophysiological states, bladder afferents generate increased input into the central nervous system for lower bladder volumes, leading to exaggerated bladder sensations and bladder dysfunction (Yoshimura et al., 2014). The complex physiological response to BCG provides multiple potential avenues for the development of bladder afferent sensitization.

### Inflammation

The therapeutic mechanism of action of BCG relies on the induction of a significant local inflammatory response within the bladder mucosa (Redelman-Sidi et al., 2014). Bladder inflammation (cystitis) is key modulator of bladder afferent hypersensitivity and is commonly associated with the development of LUTS in a number of distinct inflammatory bladder disorders including urinary tract infection and interstitial cystitis/bladder pain syndrome, and chemotherapy induced cystitis (Suh et al., 2017; Grundy et al., 2018a; Mills et al., 2020). Intravesical BCG evokes a significant localized immunological response characterized by the influx of macrophages, dendritic cells and neutrophils into the bladder wall within hours of instillation (Simons et al., 2008). CD4^+^ and CD8^+^ T cells are also found in increased numbers in the urine and mucosa of BCG-treated patients (Boccafoschi et al., 1992; de Boer et al., 1993; Ingersoll and Albert, 2013). Highly elevated levels of urinary cytokines IL-2, IL-6, IL-8, IL-18, IL-1ra, IFN-γ, IL-12[p70], TNF-a, GM-CSF and chemokines including monocyte chemoattractant protein (MCP)-1, MIP-1α, and interferon-inducible protein (IP)-10 are found following BCG treatment (Taniguchi et al., 1999; Redelman-Sidi et al., 2014). As BCG infusions continue during induction, chemokine signaling further increases the recruitment of monocytes and neutrophils and the release of pro inflammatory cytokines to sustain a significant inflammatory response that is key to BCG efficacy and has the potential to sensitize bladder-innervating afferents (Simons et al., 2008; Ingersoll and Albert, 2013). However, whilst it seems logical to assume that inflammation induced sensitization of bladder-innervating afferent nerve endings is a crucial conduit of BCG induced LUTS, no studies have so far directly investigated if BCG-induced inflammation evokes bladder afferent hypersensitivity.

### Urothelial permeability

Toxic waste metabolites within urine are prevented from accessing the underlying bladder interstitium and peripheral afferent endings by the urothelial barrier. The urothelial barrier is maintained by tight junctions between apical urothelial cells, hydrophobic uroplakin plaques, and a glycosaminoglycan mucus layer that block the movement of small molecules and urine (Jafari and Rohn, 2022). Experimentally induced urothelial permeability is a known driver of bladder afferent hypersensitivity (Grundy et al., 2020b) and the development of a bladder pain phenotype in animal models (Offiah et al., 2017). Clinically, increased urothelial permeability has been linked to the development of LUTS in interstitial cystitis/bladder pain syndrome via increasing urine access to afferent endings within the sub-urothelium (Hurst et al., 2015; Grundy et al., 2020b). There is no current experimental evidence to suggest that BCG acts directly to cause urothelial barrier breakdown, however, urothelial permeability may eventuate indirectly, as a consequence of inflammation (Grover et al., 2011; Redelman-Sidi et al., 2014; Jafari and Rohn, 2022). Long-term downregulation of uroplakins, which play an indispensable role in maintaining the urothelial barrier (Hu et al., 2002), was reported following intravesical BCG treatment in a mouse model (Saban et al., 2007). Furthermore, the elevated levels of cytokines within the urine of patients following BCG (Redelman-Sidi et al., 2014) which are presumably released from immune cells infiltrating the suburothelium and urothelium in response to BCG instillation, provides indirect evidence of increased urothelial permeability. Increased levels of urinary albumin are also reported following intravesical BCG and correlate with levels of urinary cytokines, indicating urothelial leakage in the first 12 h after BCG treatment (de Boer et al., 1993).

### Direct interactions between BCG and sensory afferents

Direct interactions between bacteria, bacterial toxins, metabolites, N-formyl peptides and sensory nerves are now well characterized in a variety of organs (Yang and Chiu, 2017; Lagomarsino et al., 2021). However, interactions between BCG or the toxins and metabolites released from BCG during intravesical instillation and the sensory nerves within the bladder wall have yet to be explored. Bladder innervating neurons express the cellular architecture required to interact directly with BCG, including pattern recognition receptors such as Toll Like Receptors (Ohadian Moghadam and Nowroozi, 2019; Acioglu et al., 2022). Additionally, bladder afferent endings are found extending into the urothelial layer in close proximity to the bladder lumen (Spencer et al., 2018). Nevertheless, it is not clear if BCG penetrates deeply enough into the urothelium to interact with the underlying afferent endings. Furthermore, the functional consequences of BCG-neuronal interactions in the bladder have yet to be investigated. A recent study showed mycobacterium strain *Mycobacterium tuberculosis* can activate nociceptive neurons via the production of the glycolipid sulfolipid-1 (SL-1) (Ruhl et al., 2020). The same study, however, showed that *Mycobacterium bovis*, from which BCG for NMIBC is derived, does not produce SL-1, nor does it induce calcium transients in an immortalized mouse sensory neuron cell line (Ruhl et al., 2020).

## Clinical management strategies for BCG induced LUTS

Management of local bladder side effects caused by intravesical BCG treatment is a key aspect to ensure treatment compliance and improve bladder cancer survivorship. Despite this, there are no specific guidelines for the management of BCG-induced LUTS outlined by the American Urological Association (AUA). The European Association of Urology (EAU) recommends the use of anticholinergics including oxybutynin and propantheline bromide, paracetamol, NSAIDS including ibuprofen, or phenazopyridine (Babjuk et al., 2022). However, there is limited clinical evidence to support these guidelines and no obvious clinical consensus on which intervention offers the best patient outcomes (Table 1).

**Table 1:** Management strategies for BCG cystitis induced LUTS.

**Target**	**Rationale**	**Drug/medication**	**Mechanism**	**Clinical evidence**
**Detrusor smooth muscle contraction**				
Kamali et al. (2020)	Decreasing detrusor muscle contractions may reduce urinary urgency and frequency. First line therapies for detrusor overactivity in OAB patients.	**Oxybutynin:** Antimuscarinic	Induces bladder relaxation through inhibition of muscarinic receptors on the detrusor smooth muscle.	Oxybutynin significantly reduced urinary urgency compared to placebo (*N* = 60, *p* < 0.001).
Johnson et al. (2013)				Oxybutynin significantly increased urinary frequency (*p* < 0.01) and burning on urination (*p* < 0.05) compared to placebo (*N* = 50)
Sun et al. (2021)		**Mirabegron:** β3-adrenoreceptor agonist	Increases cyclic adenosine monophosphate concentrations, causing relaxation of bladder smooth muscle	Mirabegron significantly reduced urinary frequency, urgency, nocturia and pain when compared to placebo (*N* = 160, *p* < 0.001)
**Inflammation**				
Kamali et al. (2020)	Acute bladder inflammation has a positive correlation with increase in urinary urgency, frequency and pain.	**Celecoxib:** COX-2 Selective NSAID	Reduced inflammation via COX-2 inhibition	Celecoxib significantly reduced urinary frequency (*p* < 0.001), urinary urgency (*p* < 0.001) and dysuria (*p* < 0.01) when compared to placebo (*N* = 60).
**Sensory Nerves**				
Kamali et al. (2020)	Peripheral endings of bladder sensory nerves become hypersensitive during BCG cystitis.	**Phenazopyridine:** urinary analgesic	Mechanism of action is still not well understood—proposed to interact directly with sensory nerves via TRPM8.	Phenazopyridine significantly reduced urinary frequency (*p* < 0.01), urinary urgency (*p* < 0.001) and dysuria (*p* < 0.001) when compared to placebo (*N* = 60).
**Bladder Permeability**				
Lee et al. (2022)	Increased bladder permeability caused by inflammation allows toxic waste products in urine to act on bladder sensory nerves, inducing bladder hyperexcitability and pain.	**Pentosan polysulphate sodium**	Assists in restoring the barrier function of the urothelium.	Patients receiving PPS were significantly less likely to discontinue BCG installations due to LUTS when compared to placebo (*N* = 217, 15.6% vs 6.3% *p* < 0.05)
Yadav et al. (2016)				Post-treatment VAS scores were significantly lower in patients receiving PPS compared to controls (*N* = 32, *P* < 0.01).
Topazio et al. (2014)		**Hyaluronic acid (HA)**	Assist in restoring and replenishing the GAG layer.	Individuals with NMIBC (*N* = 15) infused with BCG and HA reported significantly lower VAS for pain compared to those infused only with BCG (*p* < 0.05).

### Inflammation

A recent randomized controlled clinical trial showed that in a cohort of 30 NMIBC patients receiving intravesical BCG, the COX-2 selective NSAID celecoxib was effective in reducing rates of urinary urgency, urinary frequency and dysuria compared to a placebo treated group (Kamali et al., 2020). However, patients were not followed up long term and the study was underpowered to assess the impact of celecoxib on oncological outcomes. Due to the necessity of the inflammatory response to BCG for therapeutic efficacy, consideration of anti-inflammatory medications for local bladder symptoms should be done so with caution.

### Sensory nerves

As the efficacy of BCG is tied to the development of inflammation, and the LUT side effects of BCG are sensory, directly targeting the peripheral ends of bladder-innervating sensory nerves without impacting the critical components of BCG induced inflammation is an attractive target for preventing BCG induced LUTS. However, very few well-controlled trials have explored directly targeting the bladder-innervating sensory nerves that are sensitized during BCG cystitis. The most well studied intervention is phenazopyridine, a urinary tract analgesic indicated for short-term treatment of irritation in the lower urinary tract. Phenazopyridine has proven effective in relieving dysuria and pain in acute uncomplicated urinary tract infections (Petrov et al., 2020), IC/BPS (Lusty et al., 2018), and perioperatively for urological procedures (Stewart et al., 2022). High quality data on the use of phenazopyridine for BCG-cystitis induced LUTS is lacking, however, clinical guidelines and cancer survivors indicate it is commonly prescribed (Kassouf et al., 2015; Babjuk et al., 2022). Results from a recent randomized clinical trial in patients receiving BCG induction for NMIBC showed phenazopyridine significantly reduced symptoms of urinary urgency, urinary frequency, and dysuria (Kamali et al., 2020). There is no data yet available on whether phenazopyridine impacts oncological outcomes for bladder cancer patients. However, evidence that phenazopyridine exerts it's analgesic effect by directly inhibiting mechanosensitive bladder afferent nerve fibers via a TRPM8 dependent mechanism suggests this would be unlikely (Aizawa and Wyndaele, 2010; Luyts et al., 2023). Safety analysis shows phenazopyridine has a favorable profile for short term use and has been used for up to 2 months to treat LUTS associated with radiation-induced cystitis with matched incidences of adverse events to placebo (Shore et al., 2020).

Pre-clinical studies of bladder sensory signaling in health and disease have identified multiple potential therapeutic targets that are highly expressed on bladder-innervating sensory nerves, including voltage gated sodium and calcium channels, various TRP channels, and P2X receptors amongst others (Vlaskovska et al., 2001; Grundy et al., 2018b,c, 2023; Vanneste et al., 2021; Ramsay et al., 2023). Crucially, these targets are not commonly expressed on immune cells or known to influence inflammation, and have been shown to inhibit bladder sensory signaling following intravesical instillation, limiting the opportunity for side effects.

### Bladder permeability

Treatments that have been used successfully to treat IC/BPS symptoms, including pentosane polysulfate (PPS), via restoration of urothelial permeability have been proposed as promising treatments for preventing BCG induced LUTS. In a clinical cohort of 217 patients receiving BCG instillations for NMIBC, co-administration of oral PPS thrice daily effectively decreased the BCG discontinuation rate compared to placebo (15.6% vs. 6.3%) without impacting BCG efficacy (Lee et al., 2022). A separate small study showed oral PPS was effective in reducing BCG induced LUTS with a reduction in the post-treatment Visual Analog Scale (VAS) score for pain, the post-treatment OAB-V8 score, and dysuria (Yadav et al., 2016). Despite this promise, a more recent large scale clinical trial was terminated given the updated precautions on PPS and the risk of vision threatening maculopathy (NCT 03549650). Utilizing an intravesical route to restore urothelial permeability, a pilot study of 30 NMIBC patients randomized to either placebo or hyaluronic acid infused concurrently with BCG showed significantly lower VAS for pain and reduced urinary frequency in those receiving hyaluronic acid (Topazio et al., 2014). Combining hyaluronic acid and chondroitin sulfate in patients has also been shown to provide significant and durable improvement of bladder pain, urinary urgency, and urinary frequency in patients with refractory BCG-induced cystitis (Imperatore et al., 2018).

### Bladder contractions

The rationale behind targeting the bladder muscle is likely the same as that for overactive bladder syndrome (OAB), whereby detrusor overactivity is thought to be a major driver of urinary urgency and frequency (Abrams, 2003). The antimuscarinic oxybutynin is a first line therapy for overactive bladder syndrome (OAB) and is effective in relieving OAB symptoms in subsets of patients (Wagg and Cohen, 2002; Andersson, 2004). However, randomized controlled trials exploring its utility in BCG- induced LUTS are limited, and the results are inconsistent. A randomized, triple-blind, placebo-controlled trial of 60 patients undergoing BCG infusions found oxybutynin was beneficial in relieving BCG induced LUTS (Kamali et al., 2020), significantly reducing patient reported urgency and dysuria. In contrast, a randomized controlled trial of 50 BCG naïve patients given oxybutynin concurrently with BCG infusion reported increased urinary frequency and dysuria compared to patients receiving BCG with placebo (Johnson et al., 2013). Further exploration of modifying bladder contraction to relieve BCG-cystitis induced LUTS has utilized mirabegron, a β3-adrenoreceptor agonist, another first line pharmacotherapy for OAB (Wagg et al., 2020). Patients randomly assigned mirabegron showed improvements in OAB symptom score, urinary urgency, and urinary incontinence caused by BCG immunotherapy compared to daily placebo (Sun et al., 2021). Mirabegron was reported to have no significant impact on dysuria, but reduced nocturia after 6 days of treatment (Sun et al., 2021).

## Conclusions

BCG-induced LUTS are both common and debilitating. Effective management has the potential to drastically improve patient quality of life during and after bladder cancer treatment and increase treatment persistence in BCG-intolerant patients. However, the optimal approach for averting BCG-induced LUTS currently lacks a universally agreed-upon consensus. Nonetheless, recent small-scale clinical trials have demonstrated the safety and effectiveness of restoring urothelial permeability and directly targeting bladder-innervating afferents to diminish excitability. Further research is required to unravel the pathophysiological mechanisms that drive BCG-induced LUTS and develop novel and efficacious therapeutic opportunities. Currently available therapies require further studies in larger patient cohorts and the assessment of short- and long-term impacts on oncological outcomes.

## Author contributions

GB: Writing – original draft. WB: Writing – original draft. MH: Writing – review & editing. SP: Writing – review & editing. LG: Conceptualization, Funding acquisition, Investigation, Supervision, Writing – original draft, Writing – review & editing.
